# Influenza Virus Respiratory Infection and Transmission Following Ocular Inoculation in Ferrets

**DOI:** 10.1371/journal.ppat.1002569

**Published:** 2012-03-01

**Authors:** Jessica A. Belser, Kortney M. Gustin, Taronna R. Maines, Mary J. Pantin-Jackwood, Jacqueline M. Katz, Terrence M. Tumpey

**Affiliations:** 1 Influenza Division, National Center for Immunization and Respiratory Diseases, Centers for Disease Control and Prevention, Atlanta, Georgia, United States of America; 2 Southeast Poultry Research Laboratory, Agricultural Research Service, U.S. Department of Agriculture, Athens, Georgia, United States of America; Erasmus Medical Center, Netherlands

## Abstract

While influenza viruses are a common respiratory pathogen, sporadic reports of conjunctivitis following human infection demonstrates the ability of this virus to cause disease outside of the respiratory tract. The ocular surface represents both a potential site of virus replication and a portal of entry for establishment of a respiratory infection. However, the properties which govern ocular tropism of influenza viruses, the mechanisms of virus spread from ocular to respiratory tissue, and the potential differences in respiratory disease initiated from different exposure routes are poorly understood. Here, we established a ferret model of ocular inoculation to explore the development of virus pathogenicity and transmissibility following influenza virus exposure by the ocular route. We found that multiple subtypes of human and avian influenza viruses mounted a productive virus infection in the upper respiratory tract of ferrets following ocular inoculation, and were additionally detected in ocular tissue during the acute phase of infection. H5N1 viruses maintained their ability for systemic spread and lethal infection following inoculation by the ocular route. Replication-independent deposition of virus inoculum from ocular to respiratory tissue was limited to the nares and upper trachea, unlike traditional intranasal inoculation which results in virus deposition in both upper and lower respiratory tract tissues. Despite high titers of replicating transmissible seasonal viruses in the upper respiratory tract of ferrets inoculated by the ocular route, virus transmissibility to naïve contacts by respiratory droplets was reduced following ocular inoculation. These data improve our understanding of the mechanisms of virus spread following ocular exposure and highlight differences in the establishment of respiratory disease and virus transmissibility following use of different inoculation volumes and routes.

## Author Summary

Most infections with influenza virus result in respiratory disease. However, influenza viruses of the H7 subtype frequently cause ocular and not respiratory symptoms during human infection, demonstrating that the eye represents an alternate location for influenza viruses to infect humans. Using a ferret model, we studied the ability of influenza viruses to cause disease following ocular inoculation. We found that both human and avian influenza viruses could use the eye as a portal of entry to establish a respiratory infection in ferrets. Influenza viruses were also detected in ocular samples taken from ferrets during virus infection. We identified that influenza viruses spread to different tissues in ferrets when inoculated by ocular or respiratory routes, and that these differences affected the transmissibility of influenza viruses in this model. This study is the first to confirm that virus can spread from the eye to the respiratory tract in a replication-independent manner, and offers greater insight in understanding the ability of influenza viruses of all subtypes to cause human infection by the ocular route.

## Introduction

Despite reports of conjunctivitis following infection with numerous respiratory pathogens (including influenza, adenovirus, respiratory syncytial virus, and others), research investigating the role of ocular infection in virus pathogenicity and transmissibility has been underrepresented [1]–[4]. Influenza virus represents a highly transmissible respiratory pathogen, resulting in >200,000 hospitalizations in the United States annually [5]. While ocular disease is generally rare following influenza virus infection in humans, viruses within the H7 subtype have demonstrated an apparent ocular tropism, with the majority of human infections with H7 influenza viruses associated with conjunctivitis [6]. Moreover, ocular complications have been sporadically documented following seasonal, 2009 H1N1 pandemic, and avian H5N1 virus infections in humans [7]–[13].

Numerous properties allow the eye to serve as both a potential site of influenza virus replication as well as a gateway for the establishment of a respiratory infection. Similar to epithelial cells within the human respiratory tract, human ocular tissue and secreted mucins express sialic acids, the cellular receptor for influenza viruses [14]–[16]. The anatomical proximity between the eye and nasal passages, notably the linkage of both systems via the nasolacrimal duct, facilitates aqueous exchange and provides shared lymphoid tissue between these sites [17], [18]. Influenza virus can rapidly spread between ocular and respiratory tissues, as was demonstrated in a recent study which detected by RT-PCR live attenuated influenza vaccine (LAIV) in nasal washes in humans within 30 minutes of experimental ocular exposure to LAIV-containing aerosols [19].

Well-characterized mammalian models to study extraocular spread following ocular inoculation with influenza virus have been limited to the mouse [20]. The ferret, widely used to study influenza pathogenesis and transmission following intranasal inoculation, has also been recognized as an appropriate experimental model for studies involving the visual system [21]–[23]. A previous study demonstrated H7N3 virus replication in nasal, ocular, and rectal tissue following ocular inoculation in ferrets, but did not comprehensively examine the ability of multiple subtypes to use the eye as a portal of entry or examine virus transmissibility following inoculation by this route [24].

It is clear from epidemiological and laboratory data that ocular exposure to influenza virus can manifest in both ocular and respiratory disease. However, the properties that contribute towards the ocular tropism of select influenza virus subtypes, and the mechanisms of virus spread from ocular to respiratory tract tissue following ocular exposure to influenza virus, are not well understood. Here, we present a ferret model where influenza virus in a liquid suspension is placed on the surface of the eye and massaged across the surface of the eye within the conjunctival sac (ocular inoculation) to study the ability of human and avian influenza viruses to cause disease and transmit to naïve animals. We found that both human and avian influenza viruses can mount a productive virus infection following ocular inoculation, attributable to replication-independent drainage of virus inoculum from the site of inoculation to respiratory tract tissue. The viral infection following ocular inoculation was capable of causing severe and fatal disease (in the case of H5N1 virus), but was less transmissible by respiratory droplets (in the case of seasonal influenza viruses) compared with infection following inoculation by the traditional intranasal route.

## Results

### Human and avian influenza viruses are capable of mounting a productive infection following ocular inoculation in ferrets

Due to a high degree of similarity in lung physiology and distribution of sialic acids in respiratory tract tissues, the ferret model is frequently utilized to model the kinetics and severity of respiratory disease following administration of human and avian influenza viruses by the intranasal route [23], [25]. To determine if this homology extends to ocular tissue, we examined the composition of sialic acids on the ferret cornea, which represents a potential site of influenza virus replication following ocular inoculation. Staining of ferret corneal epithelial sheets with biotinylated lectins specific for α2–3 and α2–6 sialic acids revealed a predominance of α2–3-linked sialic acids with relatively weak expression of α2–6 sialic acids on the epithelial surface, an expression pattern similar to human corneal and conjunctival tissue (data not shown) [2], [20].

**Figure 1:**
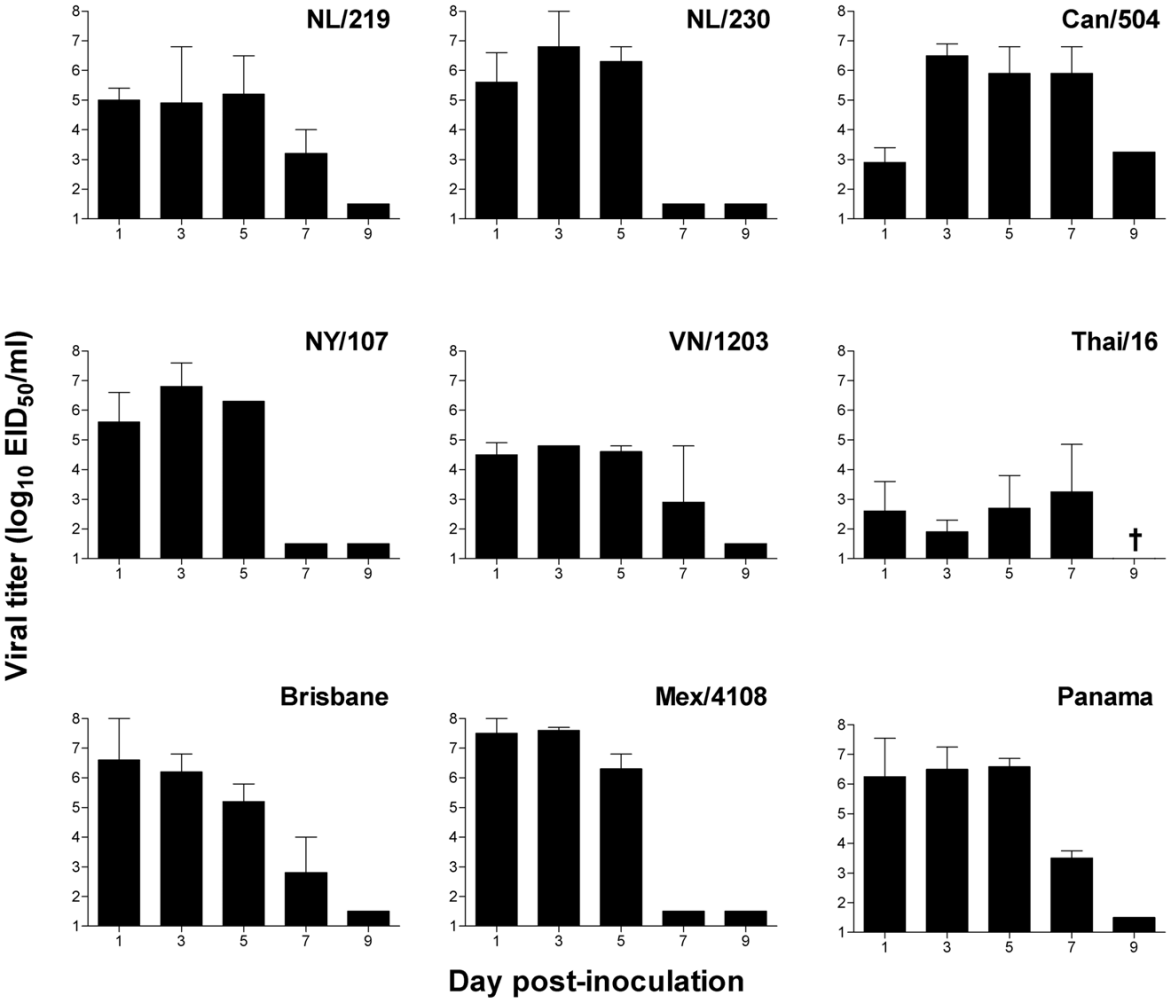
Comparison of mean titers of influenza viruses recovered from nasal wash following ocular inoculation of ferrets. Ferrets were inoculated by the ocular route with 10^6^ EID_50_/ml of each virus shown. Viral titers were measured in nasal washes collected on indicated days following serial titration in eggs; endpoint titers are expressed as mean log_10_ EID_50_/ml plus standard deviation. The limit of virus detection was 10^1.5^ EID_50_/ml. †, ferrets did not survive to day 9 p.i.

**Figure 2:**
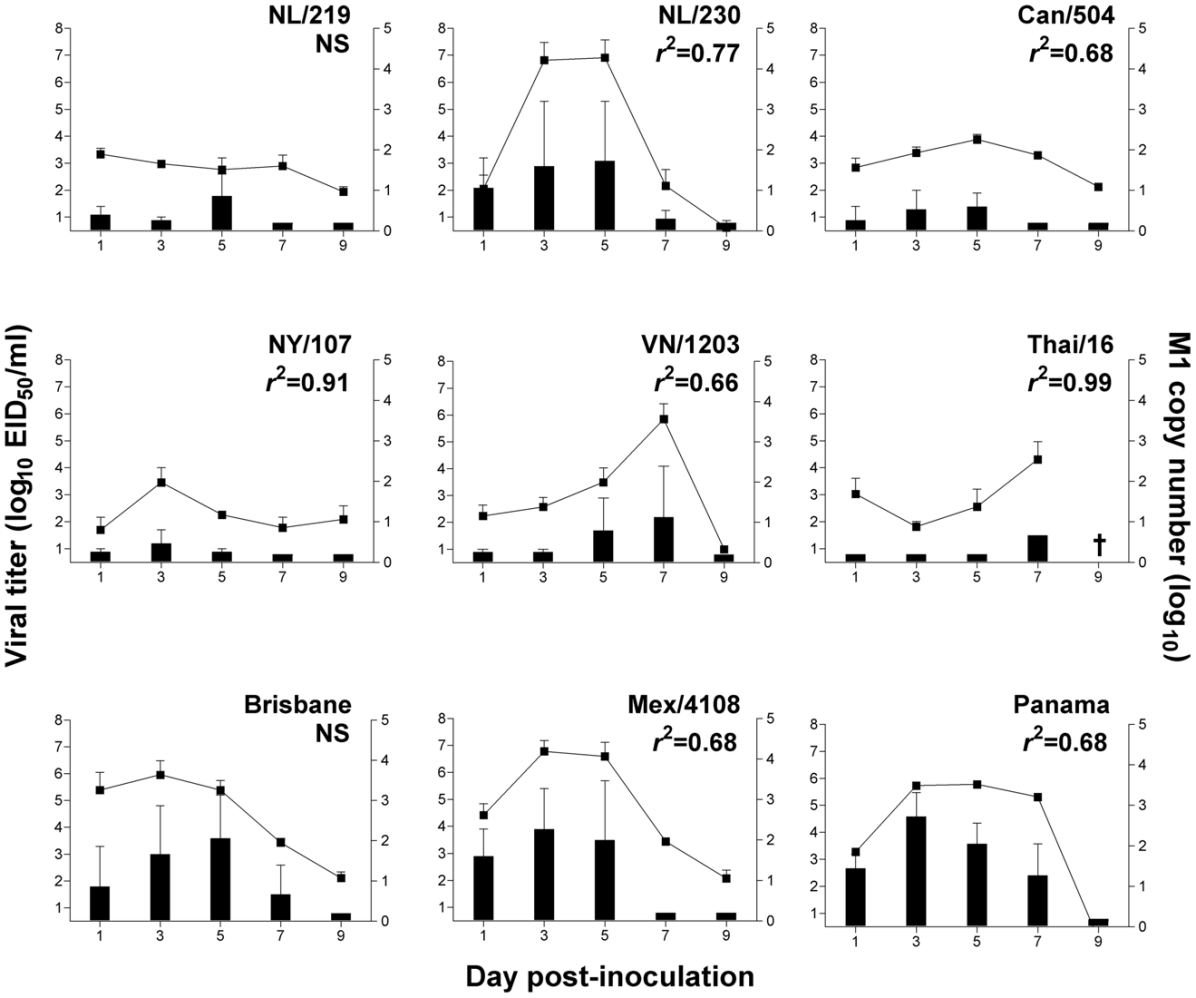
Comparison of influenza virus recovery in conjunctival wash samples following ocular inoculation of ferrets. Ferrets were inoculated by the ocular route with 10^6^ EID_50_/ml of each virus shown. Viral titers were measured in conjunctival washes (CW) collected on indicated days following serial titration in eggs; endpoint titers are expressed as mean log_10_ EID_50_/ml plus standard deviation (left y-axis and bars). Relative viral RNA copy number in conjunctival washes was determined by real-time PCR using a universal M1 primer and extrapolated using a standard curve based on samples of known virus (right y-axis and lines). The limit of virus detection was 10^1.5^ EID_50_/ml. †, no ferrets survived until day 9 p.i. R-squared values are shown for those viruses where a statistically significant (p<0.05) correlation between viral titer and viral RNA copy number exists. NS, not significant.

To assess the pathogenicity of influenza viruses of multiple subtypes following ocular inoculation (i.o.) in ferrets, we administered 10^6^ EID_50_ of each indicated virus in a volume in 100 µl on the corneal epithelial surface of the right eye of an anesthetized ferret and massaged the inoculum across the surface of the eye with the eyelid (Table 1). Ferrets were observed daily for two weeks for clinical signs of illness (including fever, weight loss, nasal or ocular discharge). Nasal wash (NW) and rectal swab (RS) samples were collected on alternate days post-inoculation (p.i.) and were titered for infectious virus, while conjunctival wash (CW) samples were collected alternate days p.i. to measure the incidence and kinetics of infectious virus replication and levels of viral RNA from inoculated eyes (Tables 1 and 2, Figures 1 and 2).

**Table 1:** Summary of virus pathotyping in ferrets following 10^6^ EID_50_ ocular inoculation.

			Following 10^6^ EID_50_ ocular inoculation in ferrets					
Virus	Name in study	Subtype^a^	# ferrets infected^b^	# ferrets survived	% mean max wt loss^c^	mean max temp change^c^	# ferrets with sneezing	# ferrets with nasal discharge
A/Netherlands/219/03	NL/219	HPAI H7N7	3/3	3/3	9.8	1.6	0/3	0/3
A/Netherlands/230/03	NL/230	HPAI H7N7	3/3	3/3	4.5	1.7	1/3	0/3
A/Canada/504/04	Can/504	HPAI H7N3	2/3	3/3	4.7	1.7	1/3	0/3
A/New York/107/03	NY/107	LPAI H7N2	3/3	3/3	2.6	1.7	0/3	0/3
A/Vietnam/1203/04	VN/1203	HPAI H5N1	2/3	2/3	25.0	2.6	2/3	2/3
A/Thailand/16/04	Thai/16	HPAI H5N1	3/3	0/3	20.1	2.4	0/3	3/3
A/Mexico/4108/09	Mex/4108	H1N1	3/3	3/3	7.4	2.2	2/3	0/3
A/Brisbane/59/07	Brisbane	H1N1	3/3	3/3	6.0	2.2	1/3	0/3
A/Panama/2007/99	Panama	H3N2	3/3	3/3	6.9	1.2	2/3	0/3

a Pathogenicity phenotype using the Intravenous Pathogenicity Index (IVPI) [60]. HPAI, highly pathogenic avian influenza; LPAI, low pathogenic avian influenza.

b As determined by isolation of virus from samples collected during observation and seroconversion at the end of the experiment.

c Average of peak mean change among ferrets from which virus was isolated from samples collected during observation.

**Table 2:** Incidence of viral replication in ferrets following 10^6^ EID_50_ ocular inoculation.

	Virus in NW		Virus in CW		Virus in RS	
Virus	# ferrets with virus in NW^a^	Peak mean titer (day)^b^	# ferrets with virus in CW	Peak mean titer (day)	# ferrets with virus in RS	Peak mean titer (day)
NL/219	3/3	5.2±1.3 (5)	3/3	1.8±0.8 (5)	3/3	2.6±1.5 (5)
NL/230	3/3	6.8±1.4 (3)	3/3	3.1±2.3 (5)	3/3	1.8±0.3 (3)
Can/504	2/3	6.5±0.4 (3)	2/3	1.4±0.5 (5)	2/3	2.0±0.7 (5)
NY/107	3/3	6.8±0.4 (3)	2/3	1.4±0.5 (3)	2/3	2.5±1.4 (5)
VN/1203	2/3	4.8±0.0 (3)	1/3	2.2 (7)	2/3	2.1±0.9 (7)
Thai/16	3/3	3.3±1.6 (7)	1/3	1.5 (7)	0/3	ND^c^
Mex/4108	3/3	7.6±1.6 (3)	3/3	3.9±1.5 (3)	3/3	2.1±0.6 (5)
Brisbane	3/3	6.6±1.6 (1)	3/3	3.6±1.6 (5)	3/3	2.3±1.3 (7)
Panama	3/3	6.6±0.3 (5)	3/3	4.6±0.9 (3)	1/3	2.25 (7)

a Limit of virus detection in nasal wash (NW) and rectal swab (RS) was 10^1.5^ EID_50_/ml, conjunctival wash (CW) was 10^0.8^ EID_50_/ml.

b Titer of ferrets with positive virus isolation expressed as log_10_ EID_50_/ml ± standard deviation.

c ND, not detected.

**Figure 3:**
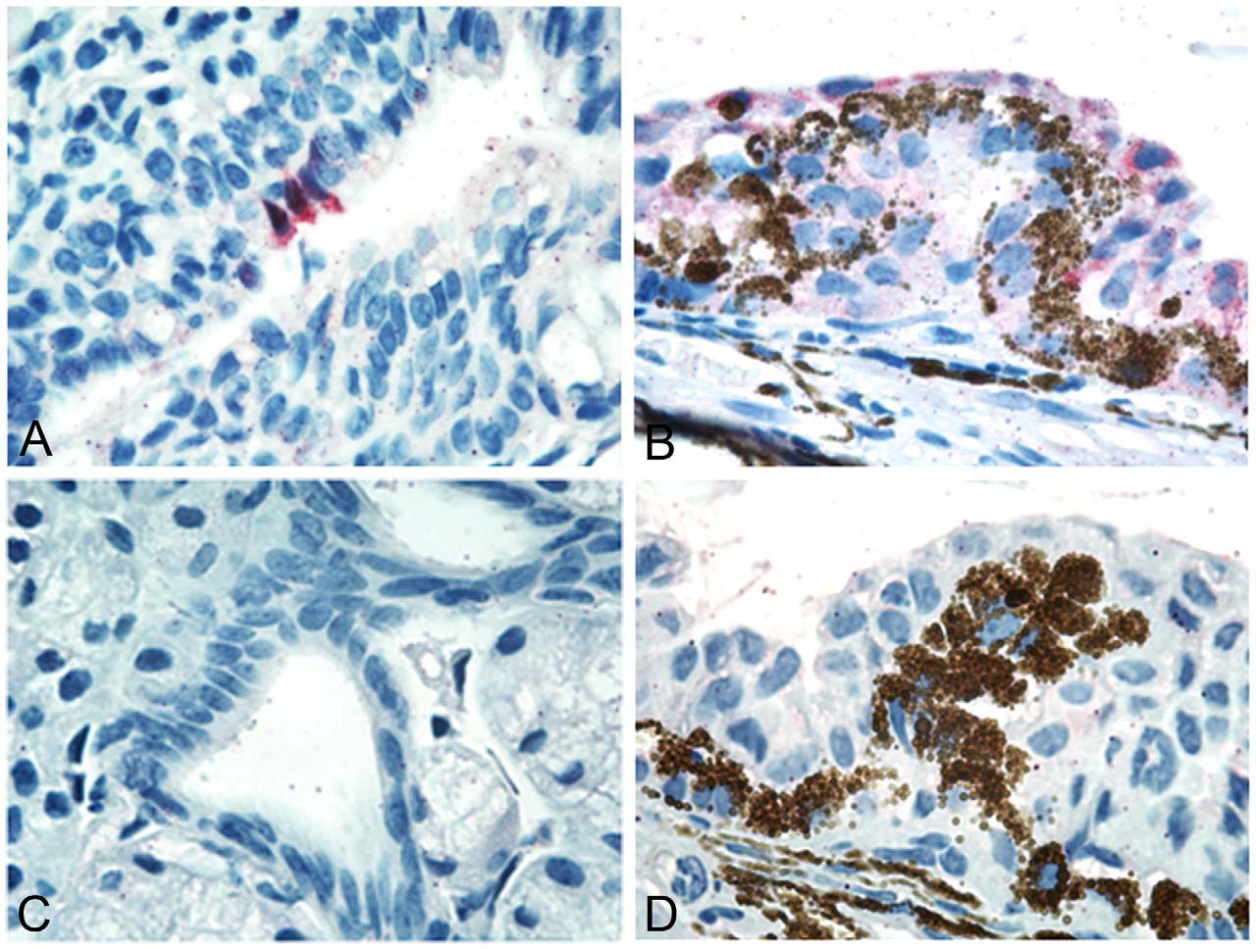
Photomicrographs of ferret tissue sections stained for the presence of influenza viral antigen following ocular inoculation. Ferrets were inoculated by the ocular route with 10^6^ EID_50_/ml of NL/230 or Brisbane virus, and tissues were collected day 3 p.i. for analysis. Viral antigen (staining in red) found in epithelial cells of lacrimal glands in the conjunctiva of a ferret inoculated with Brisbane virus (A) and epithelial cells of the ciliary processes in the eye of a ferret infected with NL/230 virus (B). No virus staining was present in the conjunctiva (C) or eye (D) of control ferrets.

All virus subtypes tested replicated in ferrets following ocular inoculation, as measured by detectable virus in NW samples as early as day 1 p.i. (Table 2 and Figure 1). The duration of virus shedding from NW samples and transient fever and weight loss generally mirrored that seen following intranasal (i.n.) inoculation for each virus [26]–[29]. However, in comparison to i.n. inoculation, the incidence of nasal discharge was reduced following i.o. inoculation with all influenza viruses tested, and sneezing was less frequent in ferrets following infection with human influenza viruses (Table 1) [30], [31]. Infectious virus was detected in CW samples from all influenza virus subtypes tested, with levels of viral RNA generally correlating with virus titers (Figure 3).

Ocular inoculation with all H7 influenza viruses tested resulted in elevated peak mean viral titers in NW samples and a higher incidence of detection in CW samples compared with H5N1 viruses (45% positive among H7 virus samples compared with 25% of H5 virus samples) (Table 2, Figure 2). However, despite reduced viral replication in NW samples, H5N1 viruses were capable of causing >20% weight loss and lethal disease in ferrets following i.o. inoculation (Table 1). Interestingly, all seasonal H1N1 and H3N2 viruses evaluated were detected at high titer in both NW and CW samples; i.o. inoculation with Mex/4108, Brisbane, and Panama viruses, along with the H7N7 NL/230 virus, resulted in the highest peak mean titers in CW samples (>10^3^ EID_50_/ml) compared with all viruses examined (Table 2, Figure 2). All viruses with the exception of Thai/16 virus were also detected at low titer in RS samples, with peak titers 10^1.8^–10^2.7^ EID_50_/ml observed days 3–7 p.i. (Table 2).

In summary, we found that both avian and human influenza viruses were capable of mounting a productive infection in ferrets following i.o. inoculation, with virus replication observed in samples collected from both ocular and respiratory tract locations regardless of virus subtype. H7 influenza viruses replicated to peak titers >2 logs higher compared with H5 influenza viruses in NW samples following i.o. inoculation, yet H5N1 influenza viruses were capable of maintaining a lethal phenotype following introduction by the ocular route. Seasonal and 2009 H1N1 pandemic influenza viruses efficiently used the eye as a portal of entry to replicate efficiently in the upper respiratory tract as well as ocular tissue.

### Intranasal and ocular inoculation routes result in differential patterns of systemic spread of virus in ferrets

To examine the capacity of influenza viruses to cause severe disease following ocular inoculation, and to better identify those features specific to ocular inoculation, we inoculated ferrets by either the traditional i.n. route (using a 1 mL inoculation volume) or the i.o. route (using a 100 µl inoculation volume) with 10^6^ EID_50_ of NL/219, NL/230, or Brisbane virus and collected systemic tissues on day 3 p.i. (Table 3 and 4). While i.n. inoculation with the H7N7 viruses tested in this study results in high virus titers throughout the respiratory tract of ferrets, H7N7 virus dissemination following i.o. inoculation was generally restricted to the upper respiratory tract, with a >3 log reduction in titers in nasal turbinates (p<0.05) and only sporadic virus isolation in trachea and lung samples compared with intranasal inoculation (p<0.005) (Table 3). A similar pattern of virus dissemination following H7N7 i.o. virus infection was observed when ferrets were inoculated by the i.n. route using a 100 µl and not 1 mL inoculation volume (Table 3). The H1N1 virus Brisbane replicated with comparable efficiency in nasal turbinate samples regardless of the inoculation route or volume chosen, but similar to H7N7 virus ocular infections, did not consistently replicate to high titers in lower respiratory tract tissues. Unlike virus dissemination to the respiratory tract, virus spread to the intestinal tract was not contingent on the route or volume of inoculation.

**Table 3:** Comparative viral pathogenesis between intranasal and ocular inoculation day 3 p.i. in extra-ocular tissue.

		Viral titer (log_10_ EID_50_/ml or g ± SD)^a^						
Virus	Route^b^	NW^c^	NT	Trachea	Lung	RS^c^	Intestine^d^	Bn OB
NL/219	i.n.	6.3±0.5 (3/3)	8.1±0.3 (3/3)	6.7±0.1 (3/3)	7.2±1.0 (3/3)	2.9±1.3 (2/3)	3.7±0.9 (3/3)	4.2 (1/3)
NL/219	i.n.^e^	5.8± 0.6 (3/3)	6.9±0.3 (3/3)	2.9 (1/3)	2.2 (1/3)	ND	4.3±1.3 (3/3)	ND
NL/219	i.o.	5.0±1.6 (4/6)	4.9±0.5 (2/2)	3.4 (1/2)	3.2 (1/2)	2.1±0.8 (4/6)	4.0±1.0 (2/2)	ND
NL/230	i.n.	5.9±0.7 (3/3)	7.4±0.8 (3/3)	6.2±1.0 (3/3)	6.8±1.0 (3/3)	2.5±0.4 (2/3)	2.4±0.4 (3/3)	3.3±0.1 (2/3)
NL/230	i.o.	6.2±1.7 (6/6)	3.6±1.6 (3/3)	ND^f^	ND	2.2±0.3 (3/6)	3.1±1.1 (2/3)	2.3 (1/3)
Brisbane	i.n.	6.1±0.5 (3/3)	5.9±0.3 (3/3)	3.7±0.6 (3/3)	ND	2.0 (1/3)	2.5 (1/3)	Not tested
Brisbane	i.n.^e^	5.2±0.4 (3/3)	6.0±0.4 (3/3)	ND	ND	ND	ND	Not tested
Brisbane	i.o.	6.7±1.1 (6/6)	6.5±1.4 (3/3)	ND	2.75 (1/3)	2.8 (1/6)	2.25 (1/3)	Not tested

a All viral titers expressed per gram of tissue except NW, NT, and RS samples which are expressed per ml. Limit of detection is 1.5 log_10_ EID_50_. The mean viral titer of all ferrets with positive virus isolation (denoted in parentheses) is shown.

b Route of inoculation. i.n., intranasal (10^6^ EID_50_/ml) unless otherwise specified; i.o.; ocular inoculation (10^6^ EID_50_/100 µl).

c i.o. NW and RS samples are inclusive of 6 ferrets tested.

d Viral titers represent a pooled intestinal sample consisting of the duodenum, jejuno-ileal loop, and descending colon.

e Intranasal inoculation performed using 10^6^ EID_50_/100 µl virus dose.

f ND, not detected.

**Table 4:** Comparative viral pathogenesis between intranasal and ocular inoculation day 3 p.i. in ocular tissue.

		Viral titer (log_10_ EID_50_/ml ± SD)^a^					
Virus	Route^b^	R eye	R conj	L eye	L conj	CW^c^	CW RNA^c^
NL/219	i.n.	3.5 (1/3)	4.1±0.5 (2/3)	3.1±0.6 (3/3)	Not tested	1.8 (1/3)	1.3±0.3 (3/3)
NL/219	i.n.^d^	ND^e^	1.0 (1/3)	ND	ND	3.5 (1/3)	0.5±0.3 (3/3)
NL/219	i.o.	2.8 (1/2)	4.3 (1/2)	ND	Not tested	1.0 (1/6)	1.5±0.5 (6/6)
NL/230	i.n.	ND	2.6±0.2 (2/3)	2.3 (1/3)	3.0±0. 4 (2/3)	ND	0.6±0.4 (3/3)
NL/230	i.o.	ND	2.75 (1/3)	ND	1.9 (1/3)	3.9±2.3 (2/6)	3.9±1.9 (6/6)
Brisbane	i.n.	ND	3.75 (1/3)	ND	2.1±0.2 (3/3)	ND	3.5±0.5 (3/3)
Brisbane	i.n.^d^	ND	1.1±0.2 (2/3)	1.3 (1/3)	1.75 (1/3)	1.25 (1/3)	2.8±0.5 (5/6)
Brisbane	i.o.	2.6±0.2 (2/3)	3.9±1.0 (3/3)	ND	2.3 (1/3)	2.9±1.7 (6/6)	3.6±0.6 (6/6)

a Limit of detection is 1.5 log_10_ EID_50_ (eye and conj) or 0.8 log_10_ EID_50_ (CW, all 100 µl i.n. samples). The mean viral titer of all ferrets with positive virus isolation (denoted in parentheses) is shown.

b Route of inoculation. i.n., intranasal (10^6^ EID_50_/ml) unless otherwise specified; i.o.; ocular inoculation (10^6^ EID_50_/100 µl).

c Relative viral RNA copy number in CW samples determined by RT-PCR and extrapolated using a standard curve of known virus. i.o. CW samples are inclusive of 6 ferrets tested.

d Intranasal inoculation performed using 10^6^ EID_50_/100 µl virus dose.

e ND, not detected.

Despite restriction of virus following i.o. inoculation to upper respiratory tract tissues compared with traditional 1 mL intranasal inoculation through day 3 p.i., virus introduced by the ocular route was still capable of causing lethal disease, as ferrets inoculated with the HPAI H5N1 virus Thai/16 by the ocular route required euthanasia days 7–8 p.i. due to development of neurological signs (Table 1). Ferrets which succumbed to Thai/16 virus infection following ocular inoculation exhibited pronounced lymphopenia in peripheral blood and systemic spread of virus to all regions of the respiratory tract and brain comparable to 1 mL intranasal inoculation, albeit with reduced lethargy and a delayed time-to-death ([32] and data not shown). These data suggest that ferrets inoculated by the ocular route succumb to a similar course of disease as intranasally inoculated ferrets, however following i.o. inoculation there is a delay in both the kinetics of virus dissemination and the development of neurological signs and severe disease, potentially owing to differences in virus inoculum reaching lower respiratory tract tissues at the time of ocular inoculation.

Ocular tissue is not routinely titrated following i.n. inoculation of influenza virus in ferrets, making it difficult to elucidate if viral titers in ocular tissue are a function of i.o. inoculation or are detected regardless of the inoculation route. Therefore, we collected both left and right whole ferret eyes and all surrounding conjunctiva/eyelid for virus titration from ferrets inoculated by the intranasal or ocular route with NL/219, NL/230, or Brisbane viruses (Table 4). Surprisingly, sporadic viral titers from both left and right eye and conjunctival tissue were detected following HPAI H7N7 virus infection by both i.n. (using either a 1 mL or 100 µl inoculation volume) or i.o. inoculation routes (Table 4). While the magnitude of viral titers and viral RNA was generally similar between intranasal and ocular routes of inoculation, real-time RT-PCR detected CW-positive samples with a greater sensitivity compared with viral culture. Isolation of virus from ocular tissue may be a reflection of the ability of these HPAI viruses to spread to extra-pulmonary tissues post-inoculation as previously described [26]. However, virus was also detected in left and right conjunctival tissue following i.n. or i.o. inoculation of the H1N1 virus Brisbane, a virus which lacks a high capacity for systemic spread [28]. Comparable levels of viral RNA were isolated from CW samples from ferrets inoculated with Brisbane virus by either intranasal or ocular routes, although infectious virus was only detected in CW samples collected from the eyes of ferrets inoculated by the ocular route. To confirm that virus detected in the eye and conjunctiva was associated with tissue-specific virus replication, immunohistochemistry (IHC) was performed to visualize the presence of influenza A nucleoprotein (NP) in ferret ocular tissues. As shown in Figure 3, influenza virus antigen was detected in epithelial cells from both the lacrimal glands in the conjunctiva and the ciliary processes in the eye collected day 3 p.i. from ferrets inoculated by the ocular route. These results indicate that the route of virus inoculation in ferrets can affect the extent of virus dissemination in respiratory tract tissue, but extra-pulmonary spread, notably to ocular tissue, is present regardless of the point of entry once an infection is established.

### Visualization of replication-independent spread of virus following ocular inoculation

**Figure 4:**
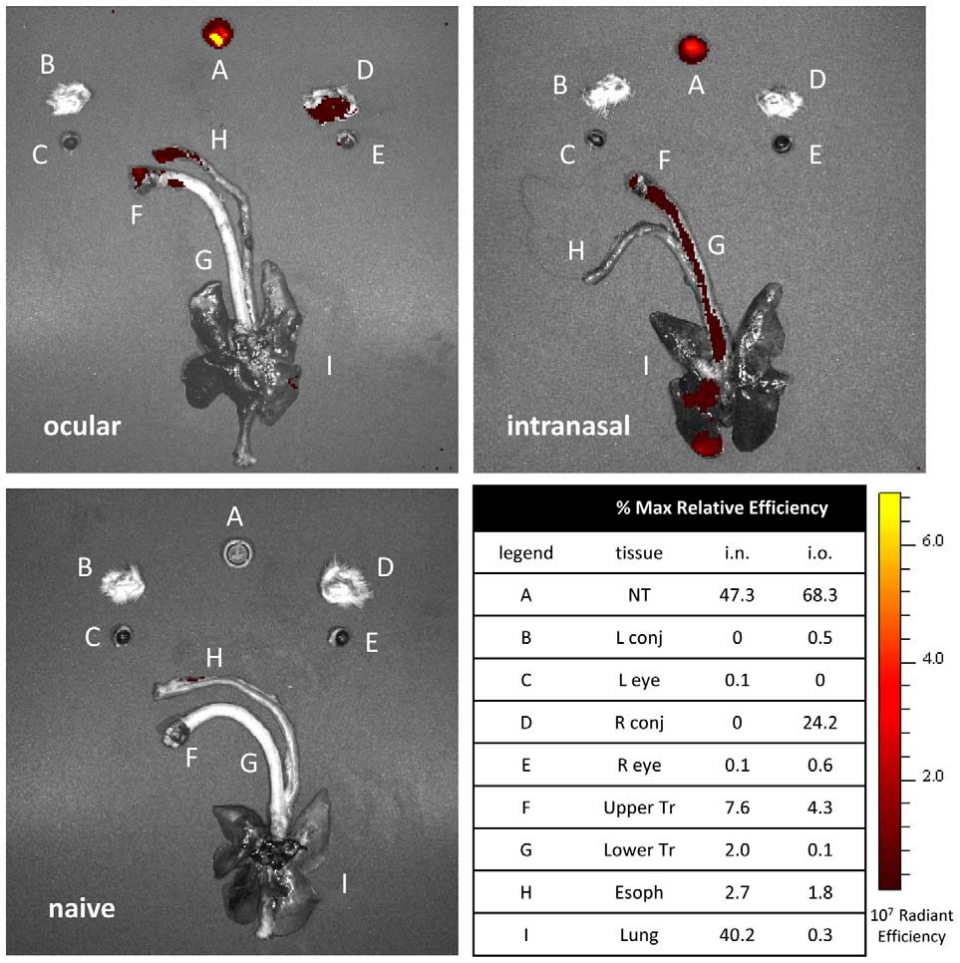
Virus deposition in ferrets following different routes of inoculation. Fluorescent-labeled A/NL/219/03 virus (NL219-FL) was administered to ferrets by the intranasal or ocular route. Each ferret was euthanized 15 min following virus administration and organs were collected for *ex vivo* imaging. Nasal turbinates are contained within the cap; left and right conjunctiva and eyes are below, respectively. An increasing fluorescence signal is indicated by brightness from red to yellow. Images are representative of triplicate independent inoculations for each route. Percentages represent the mean maximum relative efficiency for each tissue (n = 3) above levels in corresponding naïve tissue for each route of inoculation.

The detection of high viral titers in NW samples as early as day 1 p.i. following i.o. inoculation suggests replication-independent spread of virus from the eye to the respiratory tract (Figure 1); this has been similarly hypothesized in previous studies, but has yet to be proven experimentally [20], [33], [34]. Reduced viral titers in the lungs of ferrets on day 3 p.i. following ocular compared with intranasal administration further indicates differential patterns of virus spread following inoculation (Table 3). To visualize the deposition of virus immediately following different routes of inoculation, we labeled NL/219 virus with an AF680 fluorescent tag (NL/219-FL) and inoculated ferrets with equal quantities of NL/219-FL virus by the ocular (100 µl total volume) or intranasal (1 ml total volume diluted in PBS) route (Figure 4). Ferrets were euthanized 15 minutes following virus inoculation for *ex vivo* imaging. In ferrets inoculated by the traditional intranasal route, the majority of virus was deposited in the nasal turbinates and lungs, consistent with a previous study demonstrating virus dissemination throughout upper and lower respiratory tract tissue following this route of inoculation [32]. In contrast, virus deposition in ferrets inoculated by the ocular route (right side only) was primarily localized in the nasal turbinates and right conjunctiva. Lower relative quantities of virus inoculum were present in the upper trachea and esophagus following either intranasal or ocular inoculation. These findings demonstrate that, following i.o. inoculation in ferrets, influenza virus rapidly spreads to the nasal turbinates and upper trachea in a replication-independent manner, but in contrast to i.n. inoculation, does not immediately deposit in peripheral lung tissue. Furthermore, initial deposition of virus inoculum following ocular inoculation occurs not on the corneal surface of the eye but is rather concentrated in the surrounding conjunctival tissue.

### Influenza virus transmissibility following ocular inoculation

**Figure 5:**
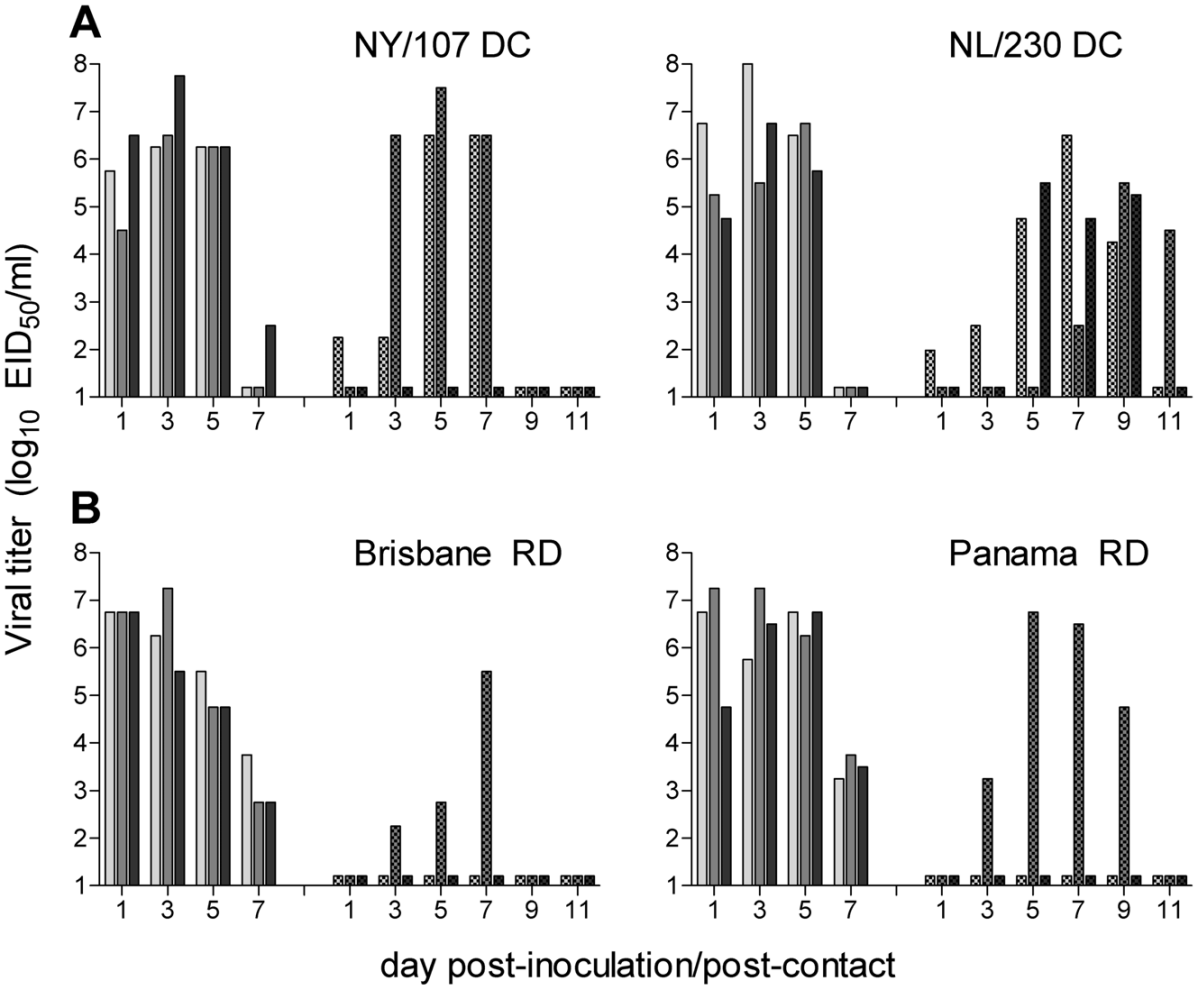
Transmissibility of influenza viruses in ferrets following ocular inoculation. Three ferrets were inoculated by the ocular route with 10^6^ EID_50_ of NY/107, NL/230, Brisbane or Panama virus, and nasal washes were collected from each ferret on the indicated day p.i. (solid bars). A naïve ferret was placed either in the same cage (A) or in an adjacent cage with perforated side walls (B) as each inoculated ferret 24 hrs p.i., and nasal washes were collected from each contact ferret on indicated days p.c. (hatched bars) to assess virus transmission in the presence of direct contact or respiratory droplets, respectively. The limit of virus detection was 10^1.5^ EID_50_/ml.

To determine if ocular exposure to influenza virus results in a transmissible respiratory infection, we inoculated ferrets by the ocular route with selected influenza viruses known to transmit following traditional i.n. inoculation to naïve contacts in the presence of direct contact or by respiratory droplets (Figure 5). Transmission was assessed by the detection of virus in NW samples and seroconversion of contact ferrets. To assess virus transmission in the presence of direct contact, ferrets were inoculated by the ocular route with the H7N2 virus NY/107 or the H7N7 virus NL/230, both viruses which transmit efficiently by this route following i.n. inoculation in ferrets [27]. Twenty-four hours later, a naïve ferret was placed in the same cage as each inoculated ferret to assess transmission. Both NY/107 and NL/230 viruses replicated efficiently in the inoculated ferrets following ocular inoculation as expected, and spread to 2/3 and 3/3 contact ferrets by day 7 post-contact (p.c.), respectively (Figure 5A). In addition to high titers of virus in the NW of contact ferrets, NY/107 contact ferrets with detectable virus in NW samples also had detectable infectious virus in CW samples (2/3 ferrets, peak titers 10^0.98–2.25^ EID_50_), and NL/230 contact ferrets had detectable infectious virus in CW (3/3 ferrets, peak titers 10^0.98–2.25^ EID_50_) and RS (2/3 ferrets, peak titers 10^1.98–2.75^ EID_50_) samples. All NL/230 and NY/107 DC contact ferrets seroconverted by the end of the observation period (data not shown). These results indicate that virus transmission in the presence of direct contact can occur following exposure to ferrets which exhibit a respiratory infection generated by i.o. inoculation, with virus recovery from contact ferrets in both respiratory and ocular samples.

To assess virus transmissibility by respiratory droplets in the absence of direct contact, ferrets were inoculated by the ocular route with the H1N1 virus Brisbane or the H3N2 virus Panama, both viruses which transmit efficiently by this route following traditional i.n. inoculation [28], [30]. Twenty-four hours following i.o. inoculation, a naïve ferret was placed in an adjacent cage with modified side walls, so that air exchange was permitted between inoculated and contact ferrets in the absence of direct or indirect contact. Unlike the efficient transmission observed with these viruses following traditional i.n. inoculation, ferrets inoculated by the ocular route with either Brisbane or Panama only transmitted virus by respiratory droplets to 1/3 contact ferrets (Figure 5B). Virus was not detected in CW or RS samples from the infected Brisbane contact ferret; the infected Panama contact ferret had a peak CW titer of 10^2.75^ EID_50_ day 5 p.c. and peak RS titer of 10^2.5^ EID_50_ day 7 p.c. While RD contacts with detectable virus in NW samples seroconverted to homologous virus at the end of the experimental period, contact ferrets which did not have detectable virus in NW samples did not exhibit seroconversion (data not shown). Ferrets inoculated intranasally with 10^6^ EID_50_ of Brisbane virus in a reduced 100 µl volume and tested for their ability to transmit virus by respiratory droplets exhibited a similar pattern of virus transmissibility as ferrets inoculated by the ocular route, with virus shedding and seroconversion detected in only 1/3 contact ferrets (data not shown). These findings suggest that despite high titers of virus in NW samples, the respiratory infection resulting from inoculation of ferrets with a reduced volume, by either ocular or intranasal inoculation routes, is distinct from that following traditional i.n. inoculation, characterized by a diminished incidence of sneezing and nasal discharge and resulting in reduced transmission of virus by respiratory droplets.

## Discussion

While the ferret has proved essential for the study of influenza virus pathogenesis and transmission, the use of this species to examine alternate inoculation methods has been limited [32], [35]–[38]. Characterizing the progression of disease following alternate routes of inoculation with influenza virus will assist in the better understanding of unique features and the relative severity and risk associated with different exposure routes. In this study, we established an *in vivo* model using the ferret to assess the ability of influenza viruses of multiple subtypes to use the eye as a gateway to establish a productive infection. Both human and avian influenza viruses were capable of mounting a respiratory virus infection in ferrets following i.o. inoculation. The detection of virus in ocular samples collected from ferrets inoculated by either ocular or intranasal routes demonstrates the importance of studying ocular involvement in respiratory virus infection. Divergent patterns of virus transmissibility by respiratory droplets following use of different inoculum volumes and routes of inoculation highlights the complexity of properties which govern virus transmission.

The high similarity of respiratory tract tissue between humans and ferrets makes the ferret model an attractive one for modeling human respiratory disease and investigating the role of receptor specificity in influenza virus pathogenesis, providing an advantage over murine models [25]. We found that the sialic acid composition of ferret corneal epithelial sheets more closely mimics that of humans compared with a mouse model, demonstrating another physiological parallel between ferrets and humans [20]. Bridging the α2–3 rich corneal and conjunctival epithelial surfaces with α2–6 rich upper respiratory tract tissue is the lacrimal duct, which expresses both α2–3 and α2–6 linked sialic acids [14], [34]. Characterization of the distribution of sialic acids in the ferret conjunctiva and lacrimal duct, in addition to the composition of ferret ocular mucins, will allow for a better understanding of virus attachment and replication in these locations. However, in a previous study, we demonstrated that the ability of influenza viruses to bind to or replicate in ocular tissue cannot be explained by sialic acid binding specificity alone [20]. Our detection in ferret ocular tissue of both human and avian influenza viruses with distinct binding specificities further underscores this point (Table 4, Figure 3).

Macroscopic signs of ocular disease in ferrets were not observed during the course of infection with any virus tested, similar to prior observations in mouse and rabbit models following deposition of influenza virus on the corneal surface [20], [39]. A previous study in ferrets reported mild conjunctival inflammation following i.o. inoculation with an H7N3 virus, however this may be attributable to strain-specific differences or the use of younger (3–5 month old) ferrets [24]. Despite the absence of visible ocular complications, virus was consistently detected in CW samples from ferrets inoculated by the intranasal or ocular route (Table 2, Figure 2). Levels of viral M1 RNA generally correlated with the magnitude of virus isolation, and were a more sensitive detection method compared with virus isolation in CW samples, similar to that observed in human eye swabs (Table 4, Figure 2) [40]. The presence of virus in RS samples following both i.n. and i.o. inoculation with influenza virus has been previously reported and likely originates from virus swallowed during inoculation [24], [32], [41], as indicated by deposition of virus in the esophagus following initial virus inoculation by both inoculation routes (Table 3, Figure 3).

Unlike in a murine model, the ferret model supported virus replication of both human and avian influenza viruses following i.o. inoculation [20]. In this ferret model, H7 viruses were detected at higher titer in NW samples and with a higher frequency in ocular CW samples compared with H5N1 viruses, suggesting a recapitulation of the tropism of the H7 virus subtype observed in humans (Table 2). However, the permissiveness of multiple virus subtypes to cause a productive infection following i.o. inoculation in the ferret points to a greater capacity of influenza viruses to use the eye as a portal of entry in an experimental *in vivo* setting, just as previous *in vitro* studies have demonstrated that numerous human ocular cell types distributed throughout the ocular area support infection and replication with both avian and human influenza viruses [42]–[44]. Cumulatively, these previous *in vitro* studies suggest that the apparent ocular tropism associated with viruses of the H7 subtype is not due to a superior ability to replicate in ocular cells compared with other virus subtypes. Future studies evaluating potential fine receptor specificity differences on the ocular surface and the composition of ocular mucins which may restrict exposure to the ocular epithelial surface to selected virus subtypes may provide a greater understanding of this property.

Consistently high titers of human influenza viruses in ocular samples following both i.n. and i.o. inoculation indicates that these H1N1 and H3N2 viruses are not exhibiting a preferential tropism for ocular infection but more likely are a reflection of the high titers observed in the upper respiratory tract in these tissues independent of the initial inoculation route. Specifically, the nasolacrimal duct which links the ocular lacrimal sac to the nasal meatus could serve as a conduit for virus-containing fluid exchange between ocular and respiratory tract tissue [18]. Numerous reports have documented drainage of vaccine or immunizing agents to nasal tissue following topical ocular administration as well as the spread of intranasally administered solutions to the conjunctival mucosal surface [18], [45]. However, spread of virus from respiratory tract tissue to ocular tissue following i.n. inoculation with human or avian influenza viruses has not been observed previously in the ferret and only sporadically reported in the mouse, possibly due to relatively low titers of virus in nasal tissue or other anatomical differences [20], [24], [26], [46]. Detection of both human and avian influenza viruses in ferret eyes and conjunctival tissue following i.n. inoculation indicates that virus can circulate proximal to the nasal cavity and nasolacrimal canal more readily than previously considered and that more routine collection of ocular tissue during standard virus pathotyping in mammalian models is warranted to better understand the extent of viral ocular dissemination (Table 4). While it is unlikely that ferret grooming practices are solely responsible for virus spread between these locations, human studies with numerous respiratory viruses have demonstrated the ability to self-inoculate between ocular and respiratory sites, and it is possible that self-inoculation could be further contributing to virus spread in this model [47], [48]. Inoculation of ferrets by the ocular route with 10^6^ EID_50_ of selected human and avian influenza viruses in a 20 µl volume resulted in comparable results to those obtained employing a 100 µl inoculation volume, indicating that replication-independent drainage of virus to respiratory tract tissue and subsequent virus detection in NW and CW samples reported in this study was not contingent on the inoculum volume (data not shown).

Visualization of virus deposition using fluorescently-tagged virus has allowed for a new understanding of dissemination patterns following *in vivo* inoculation. Using this technique, we confirmed previously hypothesized reports of replication-independent drainage from ocular to respiratory tract tissue [20], [33]. Viral load measured in respiratory tract tissues day 3 p.i. following i.n. or i.o. inoculation largely mirrored initial virus deposition patterns, with tissues possessing the greatest quantity of virus reflecting those sites of greatest initial virus deposition during virus inoculation (Table 3–4, Figure 4). Both i.o. and i.n. inoculation using a 100 µl volume resulted in detection of virus at high titers in upper and not lower respiratory tract tissues day 3 p.i., indicating that inoculation with a reduced volume leads to limited initial virus deposition in respiratory tract tissues regardless of inoculation route. The delay in high virus titer recovery from lower respiratory tract tissues during HPAI virus infection after i.o. inoculation and the delay in onset of severe disease and lethal outcome with Thai/16 virus is likely due to replication-dependent spread (and not deposition) of virus to the lower respiratory tract, suggesting that during inoculation, the majority of virus was retained in conjunctival tissues, drained to the nasal turbinates, or swallowed and diverted away from the lower respiratory tract; future study of ferret lacrimal tissue in this role is warranted. Comparable delays in onset of severe disease compared with traditional i.n. inoculation were observed in a murine model of i.o. inoculation and a ferret model of aerosol inoculation, despite ultimately similar lethal outcomes [20], [32]. The finding here of H5N1 subtype viruses using the eye as a portal of entry to initiate a lethal infection, shown previously in a mouse model, underscores the risk of ocular exposure to influenza viruses, even those subtypes not typically considered to have a tropism for this tissue [20].

Despite efficient replication of seasonal H1N1 and H3N2 viruses in the upper respiratory tract of ferrets following i.o. inoculation, these viruses did not result in frequent detection of sneezing and nasal discharge, and did not transmit efficiently to naïve contacts by respiratory droplets (Table 1, Figure 5). Infrequent sneezing is commonly observed among influenza viruses which do not transmit efficiently by respiratory droplets and could be contributing to the reduced transmissibility seen here [28], [30], [31]. Further research is needed to better understand the virologic and immunologic properties which confer the incidence of sneezing and nasal discharge and the role of these properties in virus transmissibility [49]. Additionally, the efficiency of virus transmission by respiratory droplets following i.o. inoculation was likely influenced by the reduced initial virus deposition and subsequent limited replication in the ferret trachea, as reduced virus transmissibility by respiratory droplets was observed following i.n. inoculation when using a 100 µl but not 1 mL volume (Table 3, Figure 4, and data not shown). Despite similarly high virus titers and duration of virus shedding in NW samples, the presence of expelled virus particles originating from tracheal replication which was present during traditional i.n., but not i.o. or i.n. inoculation using a reduced volume, may have contributed to differing transmission efficiencies between inoculation routes and may represent a previously unrecognized role for virus replication in tracheal tissue in virus transmissibility by respiratory droplets. In contrast, virus transmission in the presence of direct contact did not differ between inoculation routes. The reduced transmissibility of virus following i.o. inoculation is in agreement with epidemiological studies which demonstrate that the majority of human cases of conjunctivitis following H7 influenza virus exposure are self-limiting [50]. Further study evaluating the shedding of virus into the environment among persons infected with influenza viruses which cause respiratory or ocular disease will shed light on potential differences in transmission dynamics independent of virus subtype.

The diversity of potential exposures to influenza virus underscores the importance of studying the development of respiratory disease resulting from alternate exposure routes. This knowledge is critical for both a greater understanding of the establishment of influenza virus respiratory disease as well as differences in virus transmission dynamics following differing exposure routes. The facile dissemination of virus inoculum from ocular to nasal tissue, and the detection of virus in both NW and CW samples throughout the acute phase of ferret infection, highlights the ability for concurrent ocular and respiratory disease following influenza virus infection; not surprisingly, reports of conjunctivitis and influenza-like illness in the same individual have been documented during H7 outbreaks resulting in human infection [40], [51]. While much regarding the properties which regulate the ocular tropism of influenza viruses remains to be determined, our results highlight the potential for a range of influenza A subtypes to initiate infection through the eye and support the use of eye protection during occupational exposure to aerosols containing influenza viruses [19], [52], [53].

## Materials and Methods

### Ethics statement

This study was carried out in strict accordance with recommendations in the Guide for the Care and Use of Laboratory Animals of the National Institutes of Health. All ferret procedures were approved by Institutional Animal Care and Use Committee (IACUC) of the Centers for Disease Control and Prevention and in an Association for Assessment and Accreditation of Laboratory Animal Care International-accredited facility. Animal studies were performed in accordance with the IACUC guidelines under protocol #2195TUMFERC-A3: “Studies on the Pathogenesis and Transmission of Recombinant Influenza Viruses in Ferrets”.

### Viruses

Influenza A viruses of the H7, H5, and H1 subtype used in this study are shown in Table 1. Virus stocks were propagated in the allantoic fluid cavity of 10 day old embryonated hens' eggs as previously described [26]; virus stocks were confirmed by sequencing to be free of mutations. The 50% egg infectious dose (EID_50_) for each virus stock was calculated by the method of Reed and Muench [54] following serial titration in eggs. Fluorescent-tagged virus (NL/219-FL) was generated using formalin-inactivated NL/219 virus and a SAIVI Antibody Alexa Fluor 680 Labeling kit (Invitrogen) per manufacturer's instructions as previously described [32]. All experiments with HPAI viruses were conducted under biosafety level 3 containment, including enhancements required by the U.S. Department of Agriculture and the Select Agent Program [55].

### Ferret experiments

Male Fitch ferrets (Triple F Farms), 7 to 10 months old and serologically negative by hemagglutination inhibition to currently circulating influenza viruses, were used in this study. Ferrets were housed in a Duo-Flow Bioclean mobile clean room (Lab Products) for the duration of each experiment. Intranasal (i.n.) inoculations were performed under anesthesia as previously described using 10^6^ EID_50_ of virus diluted in PBS in a 1 ml or 100 µl volume [29]. Ocular (i.o.) inoculations were performed under anesthesia using 10^6^ EID_50_ of virus diluted in PBS in a 100 µl or 20 µl volume. Virus inoculum was administered dropwise to the surface of the right eye of each ferret and massaged over the surface of the eye by the eyelid.

Ferrets were monitored daily post-inoculation for morbidity and clinical signs of infection as previously described [29]. Any ferret which lost >25% of its pre-inoculation body weight or exhibited neurological dysfunction was euthanized. Virus shedding was measured on alternate days post-inoculation (p.i.) in nasal washes (NW), conjunctival washes (CW), and rectal swabs (RS). NW and RS samples were collected as previously described [28], [29]. CW were obtained by bathing the inoculated (right) ferret eye three times with 500 µl wash solution (PBS containing Pen/Strep, Gentamycin, and BSA) and collecting the run-off in a small petri dish, then swabbing the surface and surrounding conjunctival tissue of the right eye with a pre-wettened cotton swab for 5 seconds, and placing the swab in a collection tube containing the run-off liquid. All samples were immediately frozen on dry ice and stored at −70°C until processed.

To assess virus dissemination following i.n. or i.o. inoculation, three ferrets per group were inoculated with indicated viruses and euthanized 3 days p.i. for postmortem examination and collection of tissues for virus titration as previously described [29]. Tissue specimens were collected for virus titration were immediately frozen on dry ice and stored at −70°C until processed. Blood samples collected during necropsy were subjected to complete blood counts (CBCs) and serum chemistry analyses performed per manufacturer's instructions as previously described [56].

Virus transmissibility following i.o. inoculation was assessed by inoculating ferrets by the ocular route with indicated viruses and, 24 hrs p.i., placing a naïve ferret in the same cage as an inoculated ferret [to assess transmission in the presence of direct contact (DC)] or in an adjacent cage with modified side-walls to allow air exchange between inoculated and contact animals via perforations but inhibiting direct or indirect contact between animals [to assess transmission by respiratory droplets (RD)] as previously described [30]. For each i.o. transmission experiment, an aliquot of each virus stock used to characterize transmissibility in previous publications by the i.n. route was tested. NW, CW, and RS samples were collected on alternate days p.i./post-contact (p.c.) to assess kinetics of virus shedding. Serum was collected days 17–21 p.i./p.c. to measure seroconverison. Animal research was conducted under the guidance of the Centers for Disease Control and Prevention's Institutional Animal Care and Use Committee in an Association for Assessment and Accreditation of Laboratory Animal Care International-accredited animal facility.

### Sample titration and processing

NW, CW, and RS samples were serially titrated in eggs, starting at a 1∶10 dilution (NW, RS; limit of detection, 10^1.5^ EID_50_/ml) or 1∶2 dilution (CW; limit of detection, 10^0.8^ EID_50_/ml). Virus infectivity for all samples was calculated by the method of Reed and Muench [54]. At the time samples were thawed for virus titration, RNA was extracted from CW samples using a QIAamp Viral RNA kit (Qiagen). Real-time RT-PCR was performed with a QuantiTect SYBR Green RT-PCR kit (Qiagen) using an influenza A virus M1 gene primer set to determine viral load [32]. Viral RNA copy numbers were extrapolated using a standard curve based on samples of known virus as previously described [32], [57]. Baseline levels were determined by collecting CW samples from uninfected ferrets.

Tissue specimens were homogenized in 1 ml cold PBS using disposable sterile tissue grinders and clarified by centrifugation before serial titration in eggs, starting at a 1∶10 dilution. Ferret eye and conjunctival tissues were rinsed with PBS prior to virus titration. Eye, conjunctival, and nasal turbinate tissues are expressed as EID_50_/ml, while all other tissues are expressed as EID_50_/g.

### Immunofluorescence staining and microscopy

Uninfected ferret corneal epithelial sheets were dissociated from excised whole ferret eyes following incubation in tetrasodium EDTA for determination of expression of surface sialoligosaccharides as previously described [20], [58]. To assess virus dissemination, ferrets were inoculated with NL/219-FL virus either i.o. (100 µl) or i.n. (1 ml) as previously described [32]. Fifteen minutes p.i., ferrets were euthanized and respiratory and ocular tissues were excised for ex vivo imaging using a Spectrum in vivo imaging system and Living Image 4.0 Software (Caliper Life Sciences). All *ex vivo* imaging was performed in triplicate. To quantify the presence of NL/219-FL virus in excised tissues, regions of interest were drawn around each tissue using Living Image 4.0 Software to obtain maximum relative efficiency values for each tissue, expressed as photons/second/cm^2^/steradian, the mean of which was generated from three ferrets per tissue as expressed in Figure 3.

Tissues for immunohistochemistry (IHC) were collected day 3 p.i with the viruses indicated. or from uninfected ferrets, fixed by submersion in 10% neutral buffered formalin for 3 days, routinely processed, and embedded in paraffin. Immunohistochemical detection of influenza A virus nucleoprotein was performed as described previously [59].

### Statistics

The Pearson product-moment correlation coefficient was generated to measure the correlation between viral titer and viral RNA copy number in CW sample using GraphPad Prism 5.0 (GraphPad Software, Inc.). Statistical significance for all other experiments was determined using Student's *t* test.
